# Functional Analysis of the Interdependence between DNA Uptake Sequence and Its Cognate ComP Receptor during Natural Transformation in *Neisseria* Species

**DOI:** 10.1371/journal.pgen.1004014

**Published:** 2013-12-19

**Authors:** Jamie-Lee Berry, Ana Cehovin, Melanie A. McDowell, Susan M. Lea, Vladimir Pelicic

**Affiliations:** 1 MRC Centre for Molecular Bacteriology and Infection, Section of Microbiology, Imperial College London, London, United Kingdom; 2 Sir William Dunn School of Pathology, University of Oxford, Oxford, United Kingdom; Uppsala University, Sweden

## Abstract

Natural transformation is the widespread biological process by which “competent” bacteria take up free DNA, incorporate it into their genomes, and become genetically altered or “transformed”. To curb often deleterious transformation by foreign DNA, several competent species preferentially take up their own DNA that contains specific DUS (DNA uptake sequence) watermarks. Our recent finding that ComP is the long sought DUS receptor in *Neisseria* species paves the way for the functional analysis of the DUS-ComP interdependence which is reported here. By abolishing/modulating ComP levels in *Neisseria meningitidis*, we show that the enhancement of transformation seen in the presence of DUS is entirely dependent on ComP, which also controls transformation in the absence of DUS. While peripheral bases in the DUS were found to be less important, inner bases are essential since single base mutations led to dramatically impaired interaction with ComP and transformation. Strikingly, naturally occurring DUS variants in the genomes of human *Neisseria* commensals differing from DUS by only one or two bases were found to be similarly impaired for transformation of *N. meningitidis*. By showing that ComP_sub_ from the *N. subflava* commensal specifically binds its cognate DUS variant and mediates DUS-enhanced transformation when expressed in a *comP* mutant of *N. meningitidis*, we confirm that a similar mechanism is used by all *Neisseria* species to promote transformation by their own, or closely related DNA. Together, these findings shed new light on the molecular events involved in the earliest step in natural transformation, and reveal an elegant mechanism for modulating horizontal gene transfer between competent species sharing the same niche.

## Author Summary

Natural transformation is a widespread biological property in bacteria which allows them to acquire new genes. In *Neisseria meningitidis*, transformation generates an astonishing variability which contributes markedly to its success as a human pathogen. However, meningococci protect themselves from uncontrolled transformation by foreign DNA by preferentially taking up their own DNA through specific recognition of motifs known as DUS (DNA uptake sequence) by the ComP receptor. We show here that (i) ComP controls transformation in the meningococcus both in the presence and in the absence of DUS, (ii) some bases of DUS are more important for recognition by ComP and transformation, (iii) DUS variants in other human *Neisseria* commensals are impaired for transformation of *N. meningitidis*, and (iv) ComP homologs in these commensals are able to bind their cognate DUS variant and mediate DUS-specific transformation. These findings shed new light on the molecular events involved in the earliest step in natural transformation. They also reveal a previously unrecognized mechanism that is likely to play a key role in helping the many competent species that inhabit a “crowded” environment such as the human nasopharynx to curb transformation by foreign DNA and preserve species structure.

## Introduction

Natural transformation is a widespread biological property shared by dozens of bacterial species which plays a key role in evolution by generating genetic diversity through horizontal gene transfer [1]. In the human pathogen *Neisseria meningitidis*, natural transformation promotes gene reassortment on a massive scale with up to 200 genes frequently differing between isolates [2]. The meningococcus acquires DNA mainly from other meningococci but there is ample evidence for horizontal gene transfer from other bacteria sharing the same ecological niche, *i.e.* the human nasopharynx, such as other *Neisseria* species [3] or *Haemophilus* species [4]. This astonishing genome plasticity is a key virulence property of *N. meningitidis*, which contributes markedly to its success as a pathogen [5]. The same is true in other competent species where horizontal gene transfer has been implicated in the emergence of more virulent clones, and/or “superbugs” resistant to a wide variety of antibiotics.

Natural transformation, which has been studied for almost a century [6] and was key to the discovery that DNA carries genetic information [7], is thought to function as follows [8]. Free DNA is first bound by surface-exposed receptors that are likely to be components of type IV pili (Tfp) (or evolutionarily related competence pseudopili), such as the minor pilin ComP in *Neisseria* species [9]. Tfp are filamentous structures found in hundreds of species [10], which generate remarkable force upon retraction powered by the PilT ATPase [11]. Pilus/pseudopilus retraction promotes DNA translocation (or “uptake”) across formidable barriers, *i.e.* the outer membrane in Gram negative competent species [12], or the thick layer of peptidoglycan in Gram positive species [13]. Once in the periplasm/pseudoperiplasm, DNA interacts with a second widely conserved machinery that further transports it across the cytoplasmic membrane [8]. This machinery consists of another DNA receptor named ComE/ComEA [14], [15] which delivers the DNA to the permease ComEC [16] that transports it to the cytoplasm during a process powered by ATP hydrolysis [17]. Finally, bacteria become transformed upon stable, RecA-mediated incorporation of the new DNA into their genome. However, incoming DNA can also be used as a template for the repair of DNA damage (when donor is identical), or as a source of food (when donor is too different) [8].

Natural transformation by foreign DNA is a continuum between stable inheritance of a few useful new genes, and eventually loss of fitness and/or species structure, which explains why it is tightly controlled. In most competent species, transformation by foreign DNA is limited by rapid degradation by restriction enzymes and/or extensive sequence divergence that prevents RecA action [18]. However some competent species, including *N. meningitidis*, protect themselves by preferentially taking up their own DNA. It has been known for decades that uptake selectivity in *N. meningitidis*, and the closely related human pathogen *N. gonorrhoeae*, is dependent on short sequence motifs called DUS [19] that are 12 bp (ATGCCGTCTGAA) repeats representing 1% of the genome [20], [21]. In contrast, the identification of ComP as the DUS receptor is extremely recent [22]. We found that ComP, which was known to be crucial for natural transformation without affecting Tfp biogenesis [23], [24], (i) has a structure typical of type IV pilins, (ii) is required for efficient DNA binding by purified Tfp, (iii) is the only meningococcal pilin capable of binding DNA *in vitro*, and (iv) although it can bind any DNA has a marked preference for DUS [22]. Consistent with sequence conservation of DUS in *N. meningitidis* and *N. gonorrhoeae*, ComP was found to be virtually identical in these species, as well as in the human commensals *N. lactamica* and *N. polysaccharea* [22]. More distant ComP homologs were found in other *Neisseria* species and in many other members of the *Neisseriaceae* family, suggesting that ComP-mediated selective uptake of homotypic DNA is a widespread phenomenon [22]. Since canonical DUS are scarce in these latter species, many of which share the same ecological niche with *N. meningitidis* [25], it is tempting to speculate that co-evolution of DUS variants and cognate ComP receptors has occurred as a mechanism for modulating horizontal gene transfer between competent species living in the same environment.

The identification of ComP as the DUS receptor in *Neisseria* species paves the way for a detailed functional analysis of the DUS-ComP interdependence during natural transformation. Therefore, we have carried out and report here a series of experiments in which we have tested (i) the impact of ComP on transformation both in the presence and in the absence of DUS, (ii) the importance of DUS, and each of its bases, for transformation and efficient recognition by ComP, (iii) the ability of DUS variants naturally occuring in human *Neisseria* commensals to transform *N. meningitidis*, and (iv) the ability of a ComP homolog in one of these commensals (*N. subflava*) to bind its cognate DUS and mediate DUS-enhanced transformation.

## Results

### DUS enhancement of transformation in *N. meningitidis* 8013 is significant and ComP-dependent

**Figure 1:**
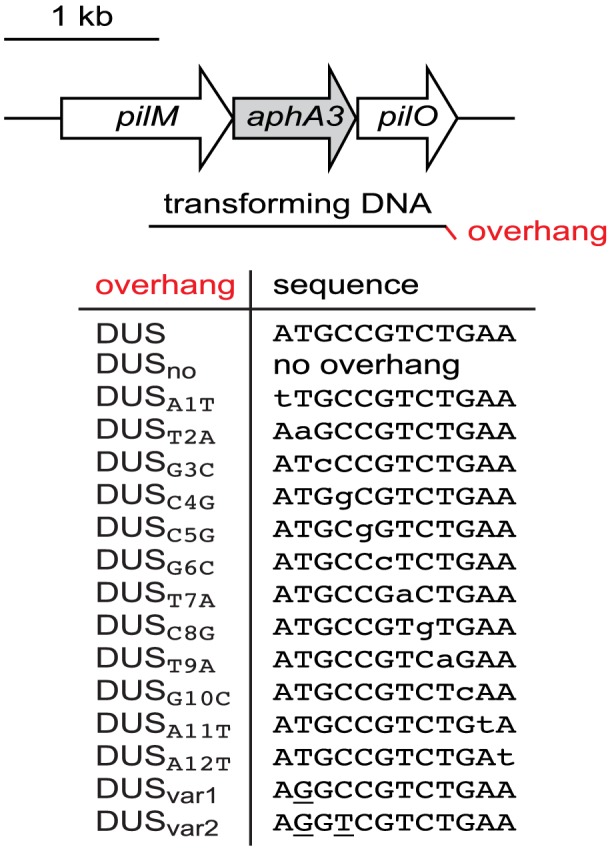
Schematic cartoon, drawn to scale, of transforming DNAs used in this study. PCR fragments (1.9 kbp) corresponding to a *ΔpilN* mutation were used for transformations. In *ΔpilN*, an *aphA3* cassette encoding resistance to kanamycin replaces the *pilN* gene and is flanked by 542 and 597 bp of *pilM* and *pilO*, respectively [29]. This fragment contains no canonical DUS (ATGCCGTCTGAA), hence DUS, or DUS mutants/variants whose base composition can be precisely controlled, can be added in 3′ by using reverse primers containing the listed overhangs.

Natural transformation has been extensively studied in multiple strains of *N. meningitidis* and *N. gonorrhoeae*. Competence has been shown to be dependent on the strain, type of DNA, and antibiotic resistance used for selection of transformants [18], [21], [26], [27]. In order to perform an in depth analysis of the functional interdependence between DUS and its ComP receptor, we designed a protocol for measuring competence in which all relevant parameters could be controlled. We focused on *N. meningitidis* clinical isolate 8013 because its genome sequence is available [2], and it is a model strain for studying Tfp biology [24], [28]. Rather than using genomic DNA for the transformation experiments, whose length and DUS composition cannot be controlled, we used a PCR fragment (1,934 bp) corresponding to a *ΔpilN* mutation in which the *pilN* gene has been replaced by an *aphA3* cassette encoding resistance to kanamycin [29]. Critically, in this PCR fragment (Figure 1) (i) the *aphA3* gene is flanked by genomic regions long enough for allelic exchange to occur efficiently and transformants to be selected [29], (ii) the GC content is similar to that of the genome [2], and (iii) no DUS with as many as two mismatches is found. Therefore, canonical or variant DUS can be added in 3′ of the transforming fragments simply by including them as overhangs (Figure 1) on the reverse primers used for PCR (Table 1).

**Table 1:** Primers used in this study.

Name	Sequence^a^
**Cloning**	
*pilV*-F	TTTCTTAATCTGCCCCGATG
*pilV*-R	TCGGCAACTTCTTTAACCAAA
*comP_sub_*-indF	cc*ttaattaa*ggagtaattttaTGCAGAGGGGCTACTCCTTG
*comP_sub_*-indR2	cc*ttaattaa*TCACCCCGTAAAAGGCCGAC
pMal-*comP_sub_*-F	gg*gaattc*CGCTCGGCCAACCTGCGTG
pMal-*comP_sub_*-R	gg*gaactt*TCACCCCGTAAAAGGCCGA
**Transformation assays**	
pilN-F1	GCCTACGCGCTATGGATAAA
pilN-R2-DUS_no_	GAGGTTCAGGATGCTGCTCT
pilN-R2-DUS	TTCAGACGGCATGAGGTTCAGGATGCTGCTCT
pilN-R2-DUS_var1_	TTCAGACGGC**c**TGAGGTTCAGGATGCTGCTCT
pilN-R2-DUS_var2_	TTCAGACG**a**C**c**TGAGGTTCAGGATGCTGCTCT
pilN-R2-DUS_T1A_	TTCAGACGGCA**a**GAGGTTCAGGATGCTGCTCT
pilN-R2-DUS_T2A_	TTCAGACGGC**t**TGAGGTTCAGGATGCTGCTCT
pilN-R2-DUS_C3G_	TTCAGACGG**g**ATGAGGTTCAGGATGCTGCTCT
pilN-R2-DUS_G4C_	TTCAGACG**c**CATGAGGTTCAGGATGCTGCTCT
pilN-R2-DUS_G5C_	TTCAGAC**c**GCATGAGGTTCAGGATGCTGCTCT
pilN-R2-DUS_C6G_	TTCAGA**g**GGCATGAGGTTCAGGATGCTGCTCT
pilN-R2-DUS_A7T_	TTCAG**t**CGGCATGAGGTTCAGGATGCTGCTCT
pilN-R2-DUS_G8C_	TTCA**c**ACGGCATGAGGTTCAGGATGCTGCTCT
pilN-R2-DUS_A9T_	TTC**t**GACGGCATGAGGTTCAGGATGCTGCTCT
pilN-R2-DUS_C10G_	TT**g**AGACGGCATGAGGTTCAGGATGCTGCTCT
pilN-R2-DUS_T11A_	T**a**CAGACGGCATGAGGTTCAGGATGCTGCTCT
pilN-R2-DUS_T12A_	**a** TCAGACGGCATGAGGTTCAGGATGCTGCTCT
**DNA-binding assays**	
DUS1^b^	TGACCATGCCGTCTGAACAAAC
DUS2	GTTTGTTCAGACGGCATGGTCA
DUS_A1T_1^b^	TGACC**t** TGCCGTCTGAACAAAC
DUS_A1T_2	GTTTGTTCAGACGGCA**a**GGTCA
DUS_T2A_1^b^	TGACCA**a**GCCGTCTGAACAAAC
DUS_T2A_2	GTTTGTTCAGACGGC**t**TGGTCA
DUS_G3C_1^b^	TGACCAT**c**CCGTCTGAACAAAC
DUS_G3C_2	GTTTGTTCAGACGG**g**ATGGTCA
DUS_C4G_1^b^	TGACCATG**g**CGTCTGAACAAAC
DUS_C4G_2	GTTTGTTCAGACG**c**CATGGTCA
DUS_C5G_1^b^	TGACCATGC**g**GTCTGAACAAAC
DUS_C5G_2	GTTTGTTCAGAC**c**GCATGGTCA
DUS_G6C_1^b^	TGACCATGCC**c**TCTGAACAAAC
DUS_G6C_2	GTTTGTTCAGA**g**GGCATGGTCA
DUS_T7A_1^b^	TGACCATGCCG**a**CTGAACAAAC
DUS_T7A_2	GTTTGTTCAG**t**CGGCATGGTCA
DUS_C8G_1^b^	TGACCATGCCGT**g**TGAACAAAC
DUS_C8G_2	GTTTGTTCA**c**ACGGCATGGTCA
DUS_T9A_1^b^	TGACCATGCCGTC**a**GAACAAAC
DUS_T9A_2	GTTTGTTC**t**GACGGCATGGTCA
DUS_G10C_1^b^	TGACCATGCCGTCT**c**AACAAAC
DUS_G10C_2	GTTTGTT**g**AGACGGCATGGTCA
DUS_A11T_1^b^	TGACCATGCCGTCTG**t**ACAAAC
DUS_A11T_2	GTTTGT**a**CAGACGGCATGGTCA
DUS_A12T_1^b^	TGACCATGCCGTCTGA**t**CAAAC
DUS_A12T_2	GTTTG**a** TCAGACGGCATGGTCA
DUS_var1_1^b^	TGACCA**g**GCCGTCTGAACAAAC
DUS_var1_2	GTTTGTTCAGACGGC**c**TGGTCA
DUS_var2_1^b^	TGACCA**g**G**t**CGTCTGAACAAAC
DUS_var2_2	GTTTGTTCAGACG**a**C**c**TGGTCA
*purL*-F^b^	GTATGGGCGGCTCGGCGTT
*purL*-R	GTCGCTGCGGTCGTGATAC

^a^ DUSs are underlined, with bases generating mutations/variants in bold lower case. Overhangs are in lower case, with restriction sites in italic.

′-labelled with biotin.^b^ Primers 5

**Figure 2:**
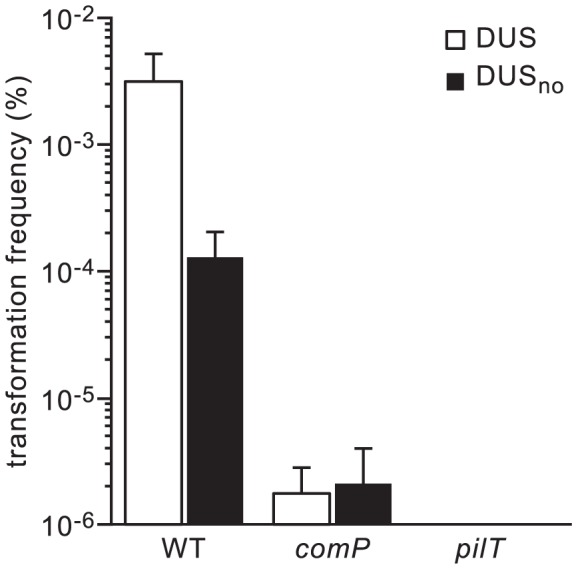
DUS enhancement of transformation in *N. meningitidis* 8013 is significant and ComP-dependent. Competence for DNA transformation in strain 8013 and its isogenic *comP* and *pilT* mutants was quantified by transforming equivalent numbers of recipient cells with 100 ng of *ΔpilN* PCR fragments obtained using primers containing the canonical DUS (white bars), or no DUS (black bars). Recipient cells and Km^R^ transformants were counted by plating. Results are expressed as percentages of recipient cells transformed, and are the mean ± standard deviation of six independent experiments.

We first quantified competence of strain 8013 in the presence and in the absence of DUS (DUS_no_) by counting the number of kanamycin-resistant (Km^R^) colonies obtained after transforming equivalent numbers of colony-forming units (CFU) with 100 ng of purified PCR fragments obtained with suitable primers (Figure 2). Km^R^ colonies were never obtained in the absence of DNA, suggesting that all the clones obtained in this assay are genuine transformants. While 0.003% recipient cells were transformed with DUS-containing DNA, mean competence was reduced almost 25 times when no DUS was present (Figure 2). This finding, which is statistically significant (*P*<0.01) as assessed by a two-tailed Student's *t* test (*P* = 0.0048), indicates that DUS enhancement of transformation in 8013 is similar to what has been described previously for other strains [26], and validates our experimental approach. We next assessed transformation frequencies in isogenic *comP* and *pilT* mutants that are dramatically impaired for competence despite being piliated [24]. In the *comP* mutant, although there was more than a 1,000-fold decrease in competence (*P* = 0.0038), few transformants were reproducibly obtained (Figure 2). Importantly, there was no DUS enhancement of transformation in this mutant since similar low frequencies of transformation were obtained regardless of the presence of DUS (Figure 2). These findings are consistent with ComP being the main DNA receptor during transformation both in the presence and in the absence of DUS. When the *pilT* mutant was tested, we found that competence was abolished (*P* = 0.0037) since no transformants could be detected (Figure 2). This finding confirms that PilT which powers pilus retraction is critical for DNA transformation [12], [24], and suggests that the background level of transformation observed in the *comP* mutant was dependent on pilus retraction, and thus Tfp-dependent.

### ComP controls transformation both in the presence and in the absence of DUS

**Figure 3:**
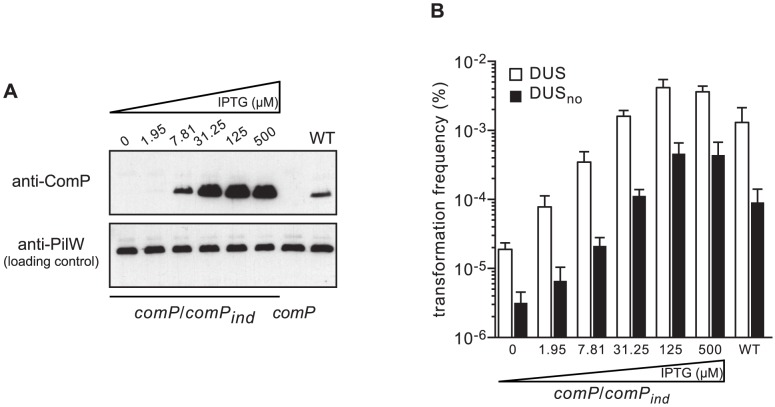
ComP controls transformation both in the presence and in the absence of DUS. (**A**) Immunoblot analysis of ComP production (upper panel) in strain *comP/comP_ind_* in the presence of increasing concentrations of IPTG. Strain *comP/comP_ind_* is a *comP* mutant complemented by an ectopic WT copy of *comP* under the control of an IPTG-inducible promoter. The WT strain was included as a control. Equal amounts of whole-cell protein extracts were loaded in each lane, which was confirmed by detecting PilW (lower panel). (**B**) Quantification of competence for DNA transformation in strain *comP/comP_ind_* in the presence of increasing concentrations of IPTG using *ΔpilN* PCR fragments obtained with primers containing the canonical DUS (white bars), or no DUS (black bars). The WT strain was included as a control. Results are expressed as percentages of recipient cells transformed, and are the mean ± standard deviation of four to six independent experiments.

Next, we assessed the impact of increasing levels of ComP on transformation by quantifying competence in a strain in which *comP* expression can be precisely controlled. In this strain *comP/comP_ind_*, a wild-type (WT) copy of *comP* under the control of an isopropyl-β-D-thiogalactopyranoside (IPTG)-inducible promoter is integrated ectopically in the genome of a *comP* mutant [24]. As assessed by immunoblotting, ComP levels increased steadily with increasing concentrations of IPTG, from undetectable levels in the absence of inducer to massive over-production above 30 µM IPTG (Figure 3A). We then quantified competence in *comP/comP_ind_* in the presence of increasing concentrations of IPTG, and in the presence or absence of DUS (Figure 3B). In the presence of DUS, transformation frequencies mirrored the results of the immunoblot analysis, with an increase in competence with increasing levels of ComP. However, WT-like competence in the *comP/comP_ind_* strain was achieved at ComP levels slightly higher than in the WT strain, which suggests that not all ComP that is produced from *comP_ind_* is functional. In the absence of IPTG, competence was low but 10-fold higher than in a *comP* mutant (see Figure 2) suggesting that some ComP was produced although it could not be detected by immunoblotting. At high concentrations of inducer, when ComP is massively over-produced, competence in the presence of DUS is significantly higher than in the WT strain (*P* = 0.0048 and 0.0016 at 125 and 500 µM IPTG, respectively) as described previously [24], [27]. These findings suggest that ComP concentration is a limiting factor for competence in a WT genetic background. Unexpectedly, when competence was quantified in the absence of DUS, a similar trend was seen (Figure 3B). There was an increase in transformation with increasing levels of ComP, with similar differences in competence between DUS and DUS_no_ at each IPTG concentration. When ComP is massively over-produced, competence in the absence of DUS is almost as high as competence in the WT in the presence of DUS (Figure 3B). Taken together, these findings show that ComP controls transformation both in the presence and in the absence of DUS, which is consistent with our finding that although ComP binds DUS preferentially, it is capable of binding any DNA efficiently [22].

### Peripheral bases of DUS are less important for recognition by ComP and transformation

**Figure 4:**
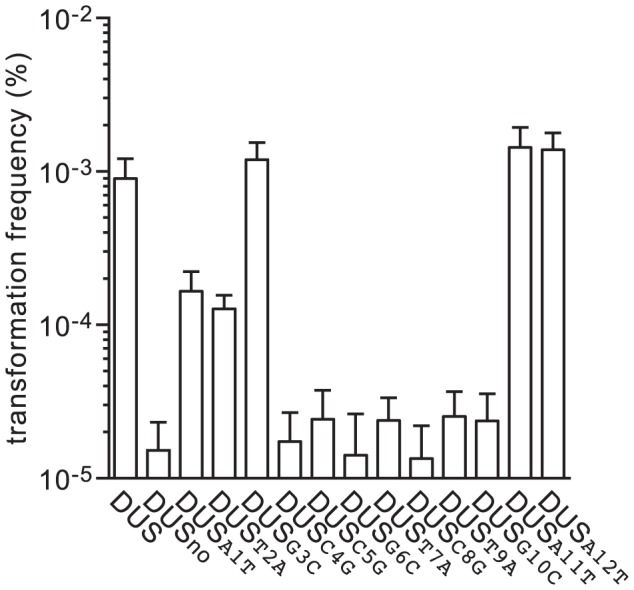
Seven inner bases of DUS are critical for enhancement of transformation. Competence for DNA transformation in *N. meningitidis* 8013 was quantified after transformation with 100 ng of *ΔpilN* PCR fragments containing a series of DUS mutants in which each base of the DUS was mutated. The specific transversion mutations are indicated in each case. As a control, the WT strain transformed with DUS and no DUS was included. Results are expressed as percentages of recipient cells transformed, and are the mean ± standard deviation of six independent experiments.

As mentioned above, DUS is highly over-represented and conserved in the genomes of *N. meningitidis* and closely related species [21]. In the genome of strain 8013 there are 1,474 copies of the canonical 12 bp DUS [2]. Interestingly, there are also 682 copies of DUS differing only by one base. It is unknown whether these mutant DUS are functional. Since the relative importance of the different bases of the DUS has never been formally assessed, we used the above method to produce transforming PCR fragments that differed only by a single base of the DUS (Figure 1). To maintain overall GC content, transversion mutations were introduced in each of the 12 bases of the DUS (Table 1), and competence was quantified (Figure 4). Perhaps unexpectedly, these experiments revealed that not all bases in the DUS are functionally equivalent. First, it confirmed that the first and second bases, which were identified only recently as being part of the DUS [20], [21], are functionally important since transformation frequencies were 5-fold (*P* = 2.06×10^−4^) and 7-fold (*P* = 1.25×10^−4^) lower when these were mutated. Surprisingly, three bases (3, 11, and 12) appeared to be dispensable since transformation frequencies with the corresponding mutants were similar to WT DUS. In marked contrast, all the other inner bases (4 to 10) were critical for DUS enhancement of transformation (Figure 4) since frequencies with the corresponding mutants were down to background DUS_no_ levels (*P*<5×10^−5^).

**Figure 5:**
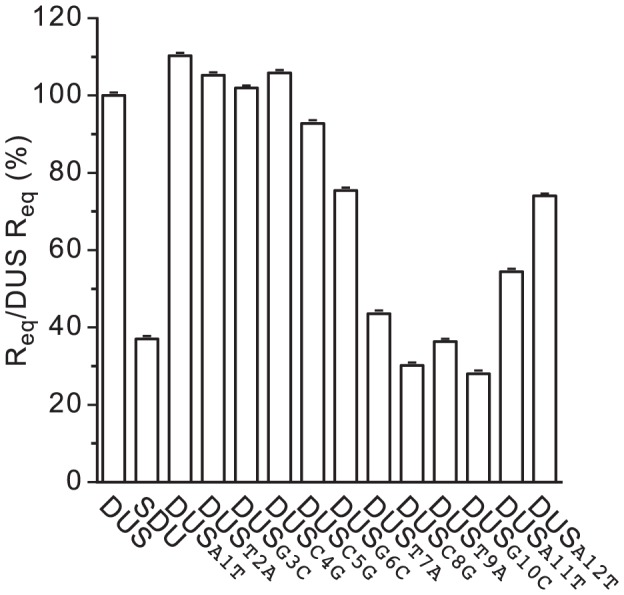
Most inner bases of DUS critical for enhancement of transformation are also critical for recognition by ComP. Similar amounts (as assessed by measuring RU) of biotinylated ds primers corresponding to DUS, SDU (a primer in which every second base of DUS is changed) and the various DUS mutants were immobilized on sensor chips. For each DNA, R_eq_ values were determined with 30 µM of pure ComP and normalized to bound RU. Results are expressed in % as ratios to DUS R_eq_, and are the mean ± standard deviation of 12 independent experiments.

In order to assess whether impaired recognition by ComP might be the reason for the observed transformation defects, we used surface plasmon resonance (SPR) as recently described [22]. We measured binding of purified ComP to double-stranded (ds) primers corresponding to each mutant DUS and compared it to binding to DUS and SDU (a scrambled primer in which every second base of DUS is changed) [22]. In brief, a neutravidin-coated sensor chip was used for immobilizing equivalent amounts of biotinylated ds primers, before 30 µM pure ComP was injected and the responses at equilibrium (R_eq_) were measured (Figure S1). This assay revealed that the inner bases of DUS are indeed important for recognition by ComP (Figure 5), with a binding as low as binding to SDU for mutants in T_7_, C_8_, T_9_ and G_10_ (*P*<5×10^−29^). Curiously, binding was less affected for mutants in C_5_ and G_6_, and apparently not affected for mutant in C_4_, although these bases were crucial for transformation. Together, these findings suggest that the inner bases of DUS (7 to 10) are critical for recognition by ComP and hence transformation.

### Transformation of *N. meningitidis* by naturally occuring DUS variants in human *Neisseria* commensals is dramatically impaired

**Figure 6:**
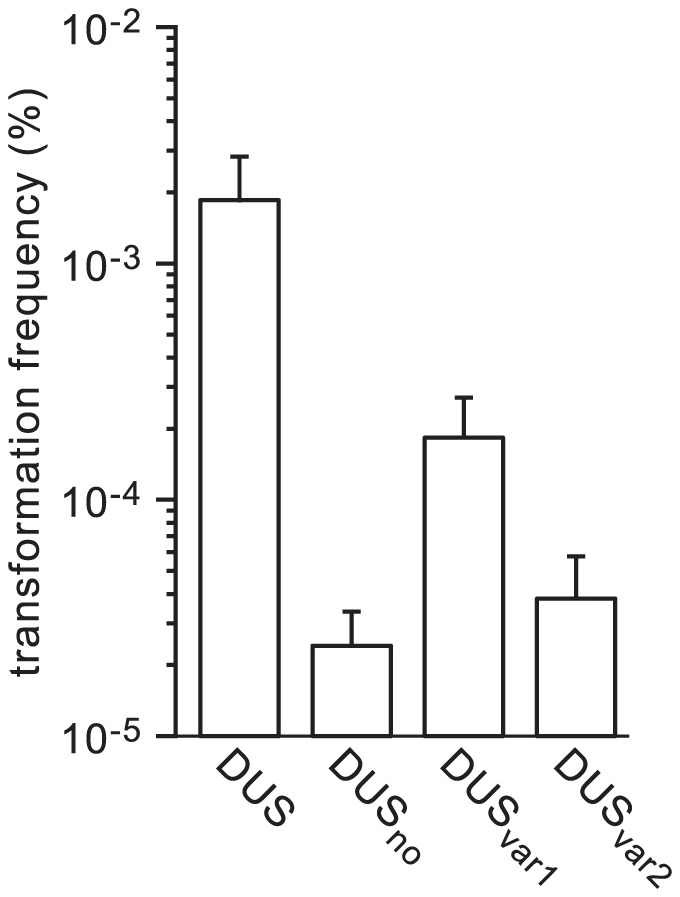
Naturally occuring DUS variants in human *Neisseria* commensals are impaired for enhancement of transformation in *N. meningitidis*. The ability of DUS_var1_ and DUS_var2_ to transform *N. meningitidis* 8013 was quantified using 100 ng of *ΔpilN* PCR fragments containing these DUS variants and compared to that of DUS and DUS_no_. Results are expressed as percentages of recipient cells transformed, and are the mean ± standard deviation of six to seven independent experiments.

Recent sequencing efforts have revealed the occurrence of gene transfers between human *Neisseria* species and, intriguingly, that at least some of these species harbour DUS variants [3]. When the genomes of the *Neisseria* species found in humans were scanned for the most frequent 12 bp repeat they contain, three variants of DUS were found. The “classical” DUS ATGCCGTCTGAA was found in *N. cinerea*, *N. gonorrhoeae*, *N. lactamica*, *N. meningitidis* and *N. polysaccharea*. A first variant differing by one base, AGGCCGTCTGAA (called DUS_var1_), was found in *N. bacilliformis*, *N. elongata*, *N. flavescens*, and *N. subflava* (and also in strain C102 which may have been erroneously identified as *N. mucosa*). A second variant differing by two bases, AGGTCGTCTGAA (called DUS_var2_), was found in *N. mucosa* and *N. sicca*. The above findings that DUS enhancement of transformation could be dramatically altered even by single base differences (see Figure 4) suggested that these minor naturally occuring variations in DUS could have a similar impact. We therefore used the above method to produce transforming *ΔpilN* PCR fragments harbouring DUS_var1_ and DUS_var2_ (Figure 1), and we tested their ability to transform *N. meningitidis* (Figure 6). The ability of DUS_var1_ (which differs from DUS by only one base) to transform strain 8013 was 10-fold lower than that of DUS, but still significantly higher than DUS_no_. In contrast, transformation by the more variable DUS_var2_ (which differs from DUS by two bases) was essentially down to levels observed in the absence of DUS (Figure 6). Intriguingly, as for some of the DUS mutants that were affected for competence but showed apparently normal binding by purified ComP (see Figure 5), SPR experiments did not reveal measurable differences in ComP binding to DUS, DUS_var1_ and DUS_var2_ (Figure S2). These results show that naturally occuring variations in DUS are expected to limit gene transfer between *Neisseria* species living in the same environment.

### The ComP homolog in the *N. subflava* commensal preferentially binds its cognate DUS and mediates DUS-enhanced transformation when expressed in *N. meningitidis*

**Figure 7:**
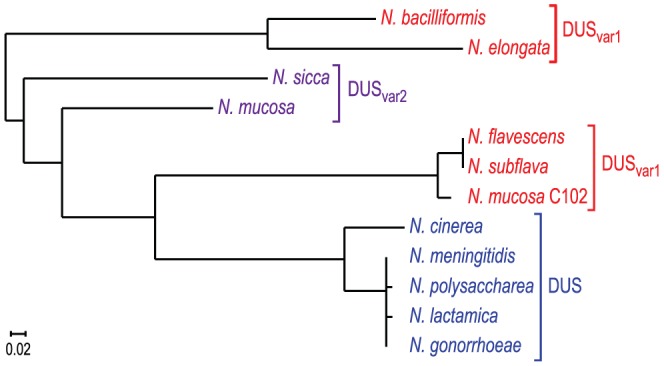
Human *Neisseria* species harbouring different DUS variants have different cognate ComPs. A maximum likelihood phylogenetic tree [36] based on the sequences of mature ComP found in human *Neisseria* species shows that species harbouring the same DUS (highlighted in the same colour) have highly conserved ComPs.

**Figure 8:**
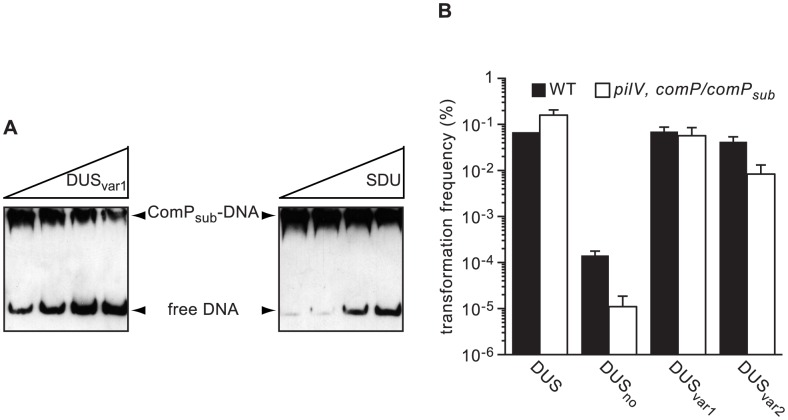
ComP homologs in other human *Neisseria* species are DNA receptors playing a similar role in transformation to meningococcal ComP. (**A**) Effect on the ComP_sub_-DNA complex of the addition of increasing amounts of unlabelled competitor as assessed by acrylamide EMSA. ComP_sub_ and biotin-labelled PCR fragment containing one copy of DUS_var1_ were incubated with two-fold increasing concentrations of unlabelled ds DUS_var1_ or SDU primers. DNA was then resolved by electrophoresis on an acrylamide native gel, transferred to a positive nylon membrane, UV-crosslinked and detected using a streptavidin-HRP conjugate. (**B**) Quantification of competence for DNA transformation in the strain 8013 (black bars) and an engineered derivative expressing ComP_sub_ (white bars). Strains were transformed in the presence of 500 µM IPTG using 10 ng of plasmids in which *ΔpilN* mutant PCR fragments containing DUS, DUS_no_, DUS_var1_ and DUS_var2_ have been cloned. Results are expressed as percentages of recipient cells transformed, and are the mean ± standard deviation of five independent experiments.

Trees derived from multiple gene phylogenies show that species harbouring the same DUS cluster together in clades [3], [25], [30], except for the species harbouring DUS_var1_ that form two distinct groups. Strikingly, a very similar tree was derived from ComP sequences only (Figure 7), showing that species habouring the same DUS have highly conserved, often virtually identical, ComPs, which extends our previous observations [22]. It was therefore tempting to speculate that these ComP homologs are DNA receptors adapted to specific recognition of their cognate DUS variants, and would play a role in competence similar to meningococcal ComP. We therefore purified ComP_sub_ from *N. subflava*, which is predicted to recognize DUS_var1_, and tested its ability to bind DNA *in vitro* using electrophoretic mobility shift assays (EMSA) as previously done for meningococcal ComP [22]. We found that ComP_sub_ has intrinsic DNA-binding ability since a shift was observed when using a biotinylated PCR fragment centered on one copy of DUS_var1_ (Figure 8A). Competition assays, during which we assessed the effect of an excess of unlabelled ds primer on the formation of ComP_sub_-DNA complex, showed that DUS_var1_ competed better than SDU as demonstrated by the gradual reappearance of the free from of biotinylated DNA (Figure 8A). This important finding confirms that ComP_sub_ has indeed a DNA-binding preference for its cognate DUS_var1_, like the one meningococcal ComP exhibits for DUS [22].

Next, we tested the functionality of ComP_sub_ *in vivo* by integrating the corresponding gene under the control of an IPTG-inducible promoter ectopically in the genome of a *N. meningitidis comP* mutant. When genomic DNA from a *N. meningitidis ΔpilN* mutant was used for transforming *comP/comP_sub_*, there was a 56-fold increase in competence (*P* = 0.003) when compared to the *comP* mutant (Figure S3), which confirms that ComP and ComP_sub_ play a similar role in competence. When a *pilV* mutation was introduced in *comP/comP_sub_*, competence was even as high as in the WT strain, which is consistent with PilV's role as an antagonist of ComP [31]. Although *pilV*, *comP/comP_sub_* could not be transformed by *ΔpilN* PCR fragments (data not shown), it could be transformed by plasmids in which these fragments PCR fragments had been cloned, which allowed us to test the effect of various DUS on transformation (Figure 8B). Surprisingly, and for an unknown reason, when plasmids were used for transformation of strain 8013 although there was a massive reduction of competence when no DUS was present (almost 500-fold), the specificity toward the various DUS was relaxed since the ability of DUS_var1_ to transform this strain was similar to that of DUS, while transformation by DUS_var2_ was only 2-fold lower. Importantly, the *pilV*, *comP/comP_sub_* strain behaved similarly (Figure 8B), with a dramatic reduction of competence when no DUS was present, similar transformation frequencies by DUS and DUS_var1_, and a significantly impaired transformation by DUS_var2_. These results confirm that DUS-specific enhancement of transformation is a property conserved among ComP orthologs.

## Discussion

Although most, if not all [32], genes involved in competence have now been identified in naturally transformable model species, our understanding of this multi-step and complex biological property is still fragmentary. Until recently, possibly the least well understood steps of natural transformation were the very first one (how the free DNA is detected and bound by Tfp/competence pseudopili) and the last one (how competent bacteria manage to preserve species structure by avoiding uncontrolled transformation by foreign DNA). The recent identification of ComP as the Tfp-exposed DNA receptor in pathogenic *Neisseria* species [22], has provided some answers to these questions. We found that *N. meningitidis* Tfp bind DNA through ComP, and that this minor pilin is at the basis of the trademark selective uptake of homotypic DNA by *Neisseria* species because of its marked preference for the signature DUS found in their genomes [21]. These findings paved the way to previously unfeasible experiments aimed at understanding the interdependence between DUS and its cognate receptor, and its impact on natural transformation.

The first important finding in this study is that ComP is a key player in natural transformation by controlling both DUS-mediated transformation and, unexpectedly, transformation in the absence of DUS. As expected, when DUS was present in the transforming DNA, there was a marked enhancement of transformation when compared to DNA without DUS. It is important to note, however, that transformation in the absence of DUS remains fairly efficient, and much higher (100-fold) than in a non-competent mutant such as *pilT*. In the absence of ComP, competence was very low and the presence of DUS did not make any difference, strengthening the notion that DUS enhancement is mediated by ComP, and suggesting that ComP is responsible for DUS_no_ transformation as well. This latter hypothesis was confirmed by the finding that overproduction of ComP resulted in increased transformation not only by DUS-containing DNA, but also by DNA devoid of DUS. Therefore, together with our previous results [22], [24], these findings suggest that ComP is the primary DNA receptor during natural transformation in *Neisseria* species, and are consistent with the following scenario. When free DNA is present, and as a result of ComP's demonstrated ability to bind any DNA [22], it is bound by *Neisseria* Tfp through ComP irrespective of the presence of DUS. Upon Tfp retraction, bound DNA is taken up and later incorporated in the genome (provided that it is similar enough to *Neisseria* DNA), as indicated by the significant transformation seen both in the presence and in the absence of DUS. However, ComP's higher affinity for DUS makes this process much more efficient with DUS-containing DNA, resulting in the well-known enhancement of transformation [21], [26] also observed here. In the absence of ComP, DNA-binding by Tfp (the mechanism for this residual binding remains to be identified) and subsequent uptake are inefficient, resulting in dramatically reduced transformation, irrespective of the presence of DUS.

The second major finding in this study is that evolution has finely tailored DUSs to their cognate ComP receptors. A mutational analysis showed that several single base substitutions in DUS (especially bases 7, 8, 9, and 10) resulted in impaired interaction with ComP and a total loss of enhancement of transformation. Nevertheless, one of the DUS mutants that was dramatically affected for competence (DUS_C4G_) was bound by ComP as efficiently as WT DUS. However, it is not impossible to imagine (among other things) that DNA binding is much tighter when ComP is within Tfp and that a minor binding defect of DUS_C4G_ undetectable *in vitro* might be significant enough *in vivo* for the bound DNA not to be taken up efficiently, which is supported by a very recent study [33] (see Note added in proof). In contrast, the peripheral bases in the DUS were found to be less important for transformation, but they are likely to play an important role (still to be identified) explaining why they are highly conserved in *Neisseria* genomes. Interestingly, a majority (72%) of the 682 copies of DUS with one base substitution that were identified in the genome of strain 8013 harbour mutations in one of the five peripheral positions and could therefore be, at least partly, functional variants. Similarly, DUS_var1_ and DUS_var2_ naturally occuring in human *Neisseria* commensals that are part of the normal flora in the nasopharynx [25], were found to be impaired for enhancement of transformation in *N. meningitidis*. Consistent with our mutational analysis, which showed that DUS_T2A_ was still partly functional for competence while DUS_C4G_ was not, DUS_var1_ that differs from DUS at base 2 was still capable of enhancing transformation significantly (although much less than DUS) while DUS_var2_ that harbours a second change at base 4 was not. However, as for DUS_T2A_ and DUS_C4G_, no ComP binding defect could be detected for DUS_var1_ and DUS_var2_ *in vitro*, but again a minor defect undetectable *in vitro* with significant consequences *in vivo* [33] remains possible. Finally, we provide evidence that ComP homologs in human *Neisseria* commensals play a similar role in transformation as indicated by the findings that ComP_sub_ from *N. subflava* binds DNA *in vitro* with a preference for its cognate DUS_var1_, and is capable of fully complementing the competence defect in a *N. meningitidis comP* mutant with a dramatic (>10,000-fold) enhancement of transformation in the presence of DUS. Surprisingly, the use of plasmids (since for an unknown reason ComP_sub_-expressing *N. meningitidis* strains are not transformable by PCR fragments) led to relaxed DUS-specificity, preventing us from demonstrating that expression of a different *comP* allele in the meningococcus leads to a switch in DUS specificity.

In conclusion, this study reveals a previously unrecognized mechanism that likely plays an important role in helping the many competent species that inhabit the human nasopharynx curb transformation by foreign DNA and preserve species structure, *i.e.* through co-evolution of DUSs and cognate pilin receptors. Although transforming DNA identity will also play an important part, it is possible to predict that *N. meningitidis* will preferably acquire genes from (in the following order) meningococci, species that share the same DUS (*N. cinerea*, *N. lactamica*, and *N. polysaccharea*), species containing DUS_var1_, species containing DUS_var2_, and then other species. Likewise, other competent *Neisseriaceae* will use their ComPs (or other unrelated pilin receptors in the *Pasteurellaceae*) in a similar fashion to promote intra-species gene transfer in a “crowded” environment such as the human nasopharynx.

### 

#### Note added in proof

While the work presented here was under review, Frye *et al.* following a DUS-centered approach reported that a variety of DUSs they nicely nicknamed “dialects” are present not only in *Neisseria* species but in most members of the *Neisseriaceae* family [33], which we previously showed harboured ComP homologs [22]. They confirmed that *Kingella denitrificans* and *Eikenella corrodens* are transformed better by DNA containing their own DUS dialect, although no selective DNA uptake could be demonstrated. Using a mutational analysis similar to ours, they found that the inner bases of the DUS are important for both DNA uptake and transformation, which we show here is due to impaired interaction with ComP. Finally, they show that different DUS dialects transform the meningococcus to various extents, which was importantly correlated to differences in DNA uptake. These findings strengthen the notion that the mechanism we have identified here is widespread.

## Materials and Methods

### Bacterial strains, plasmids and primers

*N. meningitidis* was grown on GC medium base (GCB) plates (Difco) containing Kellogg's supplements and, when required, 100 µg/ml kanamycin, 40 µg/ml spectinomycin, 3 µg/ml erythromycin, 5 µg/ml chloramphenicol (all antibiotics were from Sigma), or various concentrations of IPTG (Merck Chemicals). Plates were incubated overnight in a moist atmosphere containing 5% CO_2_. *N. meningitidis* strains used in this study have all been described. The WT strain is a derivative of the sequenced serogroup C clinical isolate 8013 [2]. In the *comP* and *pilT* isogenic mutants the corresponding genes were interrupted by the cloning of cassettes encoding resistance to spectinomycin and erythromycin, respectively [24]. Similarly, a *pilV* isogenic mutant was constructed by transforming 8013 with a pCR8/GW/TOPO-derived vector (Invitrogen) into which the *pilV* gene, amplified with *pilV*-F and *pilV*-R primers (Table 1) (all primers were from Invitrogen), had been interrupted by the cloning, in a unique *Nco*I site, of a chloramphenicol cassette extracted from pT1-Cm1 [34]. In the *ΔpilN* mutant, constructed by splicing PCR, the *pilN* gene is cleanly replaced by a kanamycin resistance cassette [29]. The *comP*/*comP_ind_* strain was obtained by transforming the above *comP* mutation in *comP_ind_* strain that contains an ectopic copy of *comP* under an IPTG-inducible promoter [24]. Using a similar approach, the *comP_sub_* allele from *N. subflava* was amplified with *comP_sub_*-indF and *comP_sub_*-indR2, cloned under an IPTG-inducible promoter and integrated in the genome of 8013, generating strain *comP_sub_*. The *comP*/*comP_sub_* and *pilV*, *comP*/*comP_sub_* strains were obtained by transforming *comP_sub_* with the above *comP* and/or *pilV* mutations.

*E. coli* DH5α was used for cloning experiments, while *E. coli* BL21(DE3) was used for protein expression and purification experiments. *E. coli* was routinely grown in liquid or solid Luria-Bertani (LB) medium (Difco) containing, when required, 100 µg/ml spectinomycin or 100 µg/ml ampicillin. Ultra-competent cells were prepared as described elsewhere [35]. The derivative of the pMAL-p2X expression vector (New England Biolabs) encoding a fusion between MBP and the soluble portion of ComP has been described previously [22]. A pMAL-p2X derivative encoding a fusion between MBP and the soluble portion of *N. subflava* ComP (ComP_sub_) was constructed in the same way. We generated pCR8/GW/TOPO-derived vectors into which various *ΔpilN* PCR fragments have been directly cloned. When doing competence assays with PCR fragments, we used the pCR8/GW/TOPO-derived vector with no DUS as a template for generating all the transforming PCR fragments. This was done to avoid “contamination” by using DUS-containing genomic DNA of the *ΔpilN* mutant as a template for PCRs. PCRs were done using the high-fidelity Herculase II Fusion DNA polymerase (Agilent). PCR fragments were purified using QIAquick PCR purification kit (QIAGEN) and quantified using a Nanodrop Lite spectrophotometer (Labtech International). When required, overhangs corresponding to DUS, mutant DUS or naturally occuring variants of DUS were added to the R2 primers (Table 1).

### Competence assays

Natural competence for DNA transformation was quantified as previously described [24]. In brief, bacteria grown overnight on agar plates were resuspended at an OD_600_ of 1 in liquid GCB containing 5 mM MgCl_2_ (called GCB transfo). When the assays were done with *comP*/*comP_ind_*, *comP*/*comP_sub_* and *pilV*, *comP*/*comP_sub_*, IPTG at desired concentrations was added into the GCB transfo. Input CFU were determined by plating serial dilutions on GCB plates containing no antibiotic. Two hundred µl of the bacterial suspension were aliquoted in 24-well plates and mixed with either 100 ng of purified *ΔpilN* PCR fragments, 200 ng of genomic DNA extracted from a *ΔpilN* mutant, or 10 ng of pCR8/GW/TOPO-derived vectors into which the various *ΔpilN* PCR fragments had been cloned. After incubating bacteria together with DNA for 30 min at 37°C on an orbital shaker (140 rpm), 0.8 ml GCB transfo was added to each well and plates were further incubated 3 h at 37°C, without shaking. Appropriate dilutions were spread on GCB plates containing kanamycin and the number of Km^R^ transformants obtained were counted the next day. Results were expressed as percentage of recipient cells transformed.

### Protein purification and DNA binding assays

ComP without its highly conserved and hydrophobic N-terminal α-helix was purified as previously described [22]. First, a MBP-trap HP column (GE Healthcare) was used to purify MBP-ComP in 50 mM sodium phosphate pH 7.5, 50 mM NaCl. After cleavage of the MBP moiety with factor Xa (New England Biolabs), the buffer was exchanged with 50 mM sodium phosphate pH 6.7, and free ComP was recovered in the same buffer containing 1 M NaCl on a HiTrap CM FF ion exchange column (GE Healthcare). ComP_sub_ was purified in a similar way.

The binding of purified ComP to various DUS sequences was measured by SPR as described previously [22], with minor modifications. Double-stranded primers corresponding to the various motifs (Table 1) were prepared by mixing complementary primers (one of which was 5′-labeled with biotin) with a 10-fold excess of the unlabelled primer, incubating the mixture at 100°C during 5 min, and slowly cooling to room temperature. Biotin-labelled double-stranded primers corresponding to the various motifs (Table 1) were coupled to neutravidin on the surface of ProteOn NLC sensor chips (BioRad), resulting in ∼230 RU increase. SPR was then performed on a ProteOn XPR36 protein interaction array system instrument (BioRad) operated at 25°C. ComP protein at 30 µM (in 25 mM HEPES pH 7.9, 150 mM NaCl, 2.5 mM MgCl_2_, 0.05% Tween-20) was then flowed over the chip surface at a flow-rate of 40 µl/min and R_eq_ were recorded. A control trace was collected using an uncoupled channel. For each DNA, we did 12 independent measurements using two separate injections of ComP over six channels.

The ability of purified ComP_sub_ to bind DNA was assessed by acrylamide EMSA as previously done for ComP [22]. In brief, we used as a target 1 fmole a biotin-labelled PCR fragment (152 bp) centered on one copy of DUS_var1_, which corresponds to an inner portion of the *purL* gene from 8013 [2]. For competition assays, two-fold increasing concentrations of unlabelled DUS_var1_ or SDU ds primers (from 22.5 to 180 pmoles) were added in a reaction containing 0.8 µM ComP_sub_ prior to the addition of biotin-labelled PCR fragment. DNA was then separated by electrophoresis on native 10% acrylamide gels, transferred to positively charged membranes and detected using the LightShift chemiluminescent EMSA kit (Pierce).

### SDS-PAGE and immunoblotting

Separation of proteins by SDS-PAGE and immunoblotting was done as described previously [29]. Gels were blotted to Amersham Hybond ECL membranes and detection was performed using Amersham ECL Prime (both from GE Healthcare).

### Bioinformatics

To identify the most over-represented sequence motifs in different *Neisseria* genomes we used the wordcount application from the EMBOSS suite (http://emboss.bioinformatics.nl). To count the number of occurrences of defined motifs in a genome, with or without mismatches, we used the fuzznuc program from the Mobyle portal (http://mobyle.pasteur.fr/). To construct a phylogenetic tree from ComPs found in human *Neisseria* species, we used the protein sequences corresponding to mature ComP (obtained by removing the short leader sequence of the corresponding pre-proteins) and the Phylogeny.fr platform (http://www.phylogeny.fr) in “One Click” mode [36].

## Supporting Information

Figure S1 Representative SPR sensograms for binding by ComP to biotinylated ds primers immobilized on four different ProteOn NLC sensor chips (BioRad). For each DNA, 30 µM pure ComP was injected and the responses at equilibrium (R_eq_) were measured. On each chip, R_eq_ from a control trace was collected using an uncoupled channel and subtracted from these values.
(EPS)

Figure S2 No difference in ComP binding between DUS and the naturally occurring DUS_var1_ and DUS_var2_ could be revealed by SPR. Similar amounts (as assessed by measuring RU) of biotinylated ds primers corresponding to DUS, DUS_var1_ and DUS_var2_ were immobilized on sensor chips. For each DNA, R_eq_ values were determined with 30 µM of pure ComP and normalized to bound RU. Results are expressed in % as ratios to DUS R_eq_, and are the mean ± standard deviation of 12 independent experiments.
(EPS)

Figure S3 Quantification of competence for DNA transformation in meningococcal strains expressing ComP_sub_. Transformations were done in the presence of 500 µM IPTG using genomic DNA from a *ΔpilN* mutant. The WT strain and *comP* mutant were included as controls. Results are expressed as percentages of recipient cells transformed, and are the mean ± standard deviation of five to six independent experiments.
(EPS)
